# Utility of PSA in extracellular vesicles as a follow-up biomarker in prostate cancer

**DOI:** 10.1515/almed-2025-0023

**Published:** 2025-07-17

**Authors:** Amaia Sandúa, José L. Pérez-Gracia, Estibaliz Alegre, Álvaro González

**Affiliations:** Service of Biochemistry, 16755 Clínica Universidad de Navarra, Pamplona, Spain; Oncology Department, Clínica Universidad De Navarra, Pamplona, Spain; IdiSNA, Navarra Institute for Health Research, Pamplona, Spain; Laboratory of Circulating Biomarkers in Cancer, Cancer Center Clínica Universidad de Navarra (CCUN), Pamplona, Spain

**Keywords:** biomarker, clinical response, extracellular vesicles, prostate-specific antigen, tumor progression

## Abstract

**Objectives:**

Prostate-specific antigen (PSA) circulates bound to extracelular vesicles (EVs). Levels of this PSA form (ev-PSA) are higher in prostate cancer (PCa) compared to benign pathologies and healthy controls, being the PSA extracellular vesicles/serum (ev/srm) ratio potentially useful as PCa diagnostic biomarker. We evaluated the utility of ev-PSA as a follow-up biomarker for detecting relapse or monitoring response to systemic treatments in advanced PCa.

**Methods:**

Samples were obtained sequentially (baseline, response and progression) from 10 patients with advanced PCa undergoing hormonal therapy or chemotherapy. EVs were isolated from serum by size exclusion chromatography. Total PSA (T-PSA) and free PSA (F-PSA) were quantified in serum and EVs in a c602 module of a Cobas 8000 (Roche Diagnostics) using Elecsys immunoassays, and PSA ev/srm ratio was calculated.

**Results:**

T-PSA in EVs (ev-T-PSA) was quantified in all samples and T-PSA ev/srm ratio median was 1.4 % (Q1-Q3: 1.1–1.9 %). At clinical response, there was not a significant decrease in ev-T-PSA (p=0.055) and neither an increase in T-PSA ev/srm ratio values (p=0.078). During progression, the T-PSA ev/srm ratio decreased significantly with respect to baseline (p=0.037) and clinical response values (p=0.008), although srm-T-PSA and ev-T-PSA concentrations did not change (p=0.625 and p=0.482, respectively). The greatest decrease in srm-T-PSA and ev-T-PSA concentrations was observed in patients undergoing hormonal therapy.

**Conclusions:**

T-PSA ev/srm ratio could be useful for detecting tumor progression and relapses in advanced PCa. However, its utility as a follow-up biomarker for assessing clinical response to hormonal treatments and chemotherapy would be limited.

## Introduction

Prostate cancer (PCa) is the second most commonly diagnosed malignancy and the fifth leading cause of cancer-related mortality among men worldwide [1], 2]. Clinically, PCa presents as a highly heterogeneous disease. While the majority of patients develop a slow-growing tumor confined to the prostate and/or achieve long-term survival due to an effective treatment, a subset of cases exhibit a more aggressive phenotype, characterized by metastatic progression and poor clinical outcomes [3].

Prostate-specific antigen (PSA) is the gold-standard biomarker of PCa. PSA is a 30 kDa serine protease synthesized by epithelial cells of the prostate gland [4], whose quantification in blood serves as a key tool for both early detection and diagnosis of PCa, given that elevated PSA levels are associated with an increased risk of disease. However, despite its specificity for prostatic tissue, PSA lacks cancer specificity, as its concentrations can also rise in benign conditions such as benign prostatic hyperplasia (BPH) or prostatitis, thereby limiting its utility as a PCa tumor marker [5], 6]. Additionally, multiple factors – including age, prostate volume, urological procedures, drugs, and others – can influence serum PSA concentration [7], [8], [9]. To enhance PSA specificity and improve diagnostic accuracy, various strategies have been explored, ranging from the development of PSA-related parameters (e.g., PSA velocity, free PSA index) to the identification of novel biomarkers and the use of multivariate models (e.g., PHI, 4Kscore test, PCA3) [10], [11], [12]. However, while some of these biomarkers have demonstrated promising potential, their clinical utility remains inconsistent, and further research to refine diagnostic approaches and identify more reliable alternatives is still needed.

PSA is a highly sensitive biomarker for disease monitoring in patients undergoing radical therapies [13], 14]. Following a successful radical prostatectomy, PSA levels should be undetectable, with any detectable concentration being indicative of poor prognosis [15]. A subsequent rise in PSA after undetectable concentrations signals biochemical recurrence [16], and a post-surgical PSA value exceeding 0.4 μg/L predicts an increased risk of new metastases [17]. PSA monitoring is also widely used to assess systemic treatments response in advanced PCa, including hormonal therapy, chemotherapy, and immunotherapy [18]. However, standardized definitions of response and disease progression remain to be established. In patients with castration-resistant metastatic PCa undergoing chemotherapy or immunotherapy, changes in PSA levels have demonstrated limited predictive value for overall survival [19], 20].

Extracellular vesicles (EVs) are small lipid membrane vesicles secreted by nearly all cells into the extracellular space, playing a crucial role in intercellular communication across multiple physiological and pathological processes [21], 22]. EVs act as carriers of parent cell-specific biomolecules, such as proteins, miRNAs, mRNAs, lncRNAs, and lipids, which can modulate signaling pathways in recipient cells [23]. Active secretion of EVs appears to be increased in cancer cells, where they contribute to multiple tumor-associated processes [24]. EVs are present in multiple biological fluids, such as blood and urine, that can be obtained through minimally invasive procedures [25], making them promising candidates as clinical biomarkers for diagnosis, prognosis, and disease monitoring in several pathologies, among them PCa [26].

Several studies have reported the expression of PSA in prostate-derived EVs [27], 28] and highlighted its potential utility as a diagnostic biomarker [29]. Logozzi et al. [30] observed significantly higher levels of plasma PSA+ CD81+ EVs in PCa patients compared to both BPH and healthy individuals, suggesting their utility in PCa screening and early diagnosis. In previous research, we also identified higher concentrations of PSA bound to EVs (ev-PSA) in PCa patients relative to BPH and healthy controls [31]. Furthermore, we demonstrated superior diagnostic performance of ev-PSA – particularly the PSA ev/srm ratio – when compared to the serum free PSA index, which is routinely used in clinical practice. Beyond diagnosis, EVs and their molecular cargo may also hold promise for PCa staging, prognosis, and monitoring of disease progression and therapeutic response [26]. Additionally, liquid biopsy biomarkers, such as AR-V7 measured in circulating tumor cells (CTCs) [32], 33] and circulating tumor DNA in castration-resistant patients [34], have been investigated for predicting resistance to hormonal therapy in advanced PCa.

In this study, we investigated the impact of different PCa therapies on PSA release in EVs, and evaluated the potential of ev-PSA as a follow-up biomarker for detecting relapse or monitoring response to systemic treatments. To achieve this, we analyzed sequentially collected samples from patients with advanced PCa undergoing different therapeutic alternatives.

## Materials and methods

### Samples and patients selection

We selected 10 patients with advanced prostate adenocarcinoma from the Department of Medical Oncology who had received hormonal therapy or chemotherapy (Table 1). According to a protocol approved by the Ethics Committee of the University of Navarra (code 2010.111), sequential samples were collected after informed consent at different times during the course of the disease: at baseline and at progression to therapy for all participants, and additionally at clinical response to treatment before disease progression in eight of these patients. The average time between baseline samples and clinical response was 4 months, while progression samples were collected 13 months after treatment initiation. The only exception was patient 2, whose progression sample was obtained 50 months after baseline. The study was conducted in accordance with the ethical principles for medical research outlined in the Helsinki Declaration.

**Table 1:** Clinical characteristics of the participants of the study. Age data are reported as median and interquartile range.

n	10
Age, years	68 (65–71)
Gleason	
≤7	3
>7	5
Unknown	2
ISUP	
<3	2
≥3	5
Unknown	3
Stage	
III	2
IV	8
Treatment	
Hormonal therapy	7
Chemotherapy	3

Blood samples were collected into 5 mL BD Vacutainer serum collection tubes (Beckton Dickinson, East Rutherford, USA). To obtain serum, tubes were centrifuged at 2000×*g* for 10 min after clotting formation. Serum samples were then aliquoted and stored at −80 °C until further analysis.

The determination of histological type, Gleason and ISUP score, clinical stage and treatments were obtained from the patients’ medical records and were based on clinical, analytical and imaging tests according to current clinical guidelines.

### Extracellular vesicles isolation

EVs were isolated from serum by Size Exclusion Chromatography using the commercial Exo-spin^TM^ mini columns kit (Cell Guidance System, Cambridge, UK). Upon thawing, serum samples were centrifuged at 16,000×*g* for 30 min. 100 μL of EVs-containing supernatant were collected and applied to the Exo-spin column previously stabilized and prepared with phosphate buffer saline (PBS). The EVs fraction was finally eluted from the column with 180 μL of PBS and diluted to a final volume of 200 μL. The dilution correction factor was taken into account in the calculation of final concentrations of PSA.

This isolation method was previously validated for the recovery of serum EVs, and the characterization of the isolated EVs was reported in a prior publication [31].

### PSA quantification

In both serum (srm-) and isolated EVs (ev-), concentrations of total PSA (T-PSA) and free PSA (F-PSA) were determined in a c602 module of a Cobas 8000 (Roche Diagnostics, Basel, Switzerland) using the commercial electrochemiluminiscent immunoassays Elecsys^®^ total PSA and Elecsys^®^ free PSA, designed for serum quantifications. The detection limits were 0.010 μg/L for T-PSA and 0.016 μg/L for F-PSA, and quantification limits 0.014 μg/L and 0.018 μg/L, respectively.

T-PSA and F-PSA ev/srm ratios (%) were calculated as follows:

T-PSA ev/srm ratio (%)=ev-T-PSA/srm-T-PSA × 100.

F-PSA ev/srm ratio (%)=ev-F-PSA/srm-F-PSA × 100.

### Statistical analysis

Statistical analysis was performed with Graphpad Prism version six using non-parametric methods. Data were represented as median and interquartile range. For comparisons, Mann–Whitney U and Wilcoxon tests were used, while for correlations Spearman’s test was performed A two-tailed p-value of <0.05 was considered to be statistically significant.

## Results

### Extracellular vesicles PSA quantification

The median of srm-PSA concentration was 14.8 μg/L for T-PSA (Q1-Q3: 6.0–109.3 μg/L) and 4.0 μg/L for F-PSA (Q1-Q3: 0.7–23.7 μg/L). Ev-T-PSA was quantified in all specimens with a median of 0.177 μg/L (Q1-Q3: 0.102–1.006 μg/L), while ev-F-PSA was detected in 96 % of the samples analyzed with a median of 0.074 μg/L (Q1-Q3: 0.034–0.224 μg/L). The values of ev/srm ratio calculated were similar for T-PSA (median 1.4 %; Q1-Q3: 1.1–1.9 %) and F-PSA (median 1.9 %; Q1-Q3: 0.8–4.6 %).

A significant correlation was observed between serum and EVs PSA concentrations for both T-PSA (r=0.958; p<0.001) and F-PSA (r=0.879; p<0.001).

### Serum and extracellular vesicles PSA analysis in sequential samples

**Figure 1:**
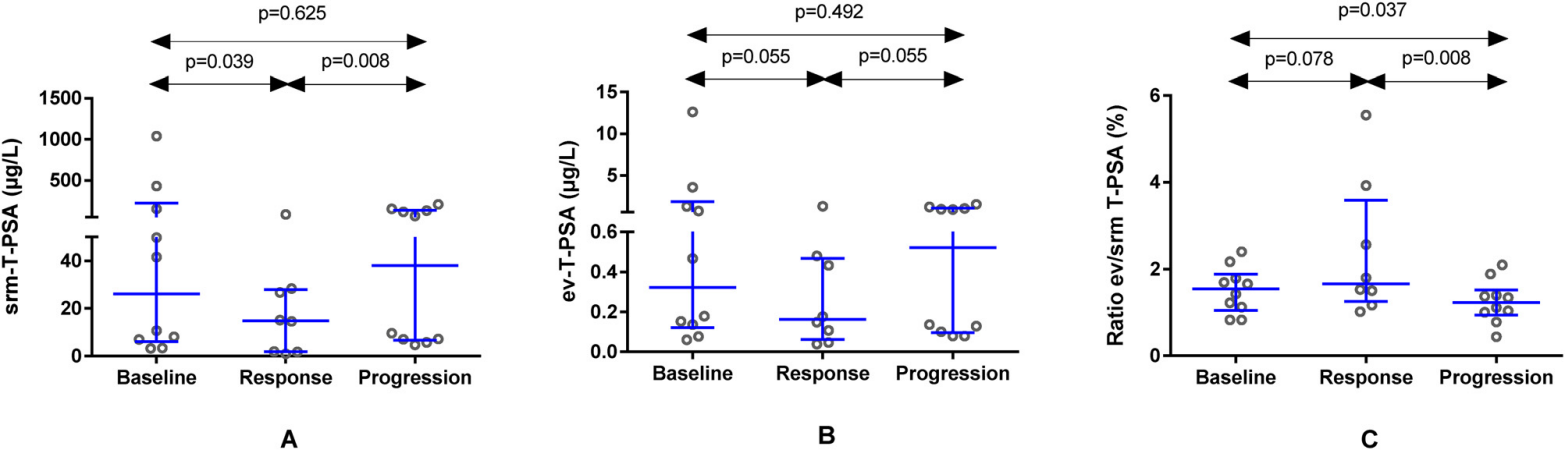
Comparative analysis of T-PSA concentrations in serum (srm−) (A) and extracellular vesicles (ev−) (B), and of T-PSA ev/srm ratio values (C) at baseline, during response to therapy and at disease progression in 10 patients with advanced prostate cancer. T-PSA, PSA total.

First, when analyzing T-PSA at clinical response, serum concentrations decreased significantly from baseline, from an initial median of 26.1 μg/L (Q1-Q3: 6.1–222.7 μg/L) to 14.8 μg/L (Q1-Q3: 1.8–27.9 μg/L; p=0.039) (Figure 1A). However, the observed decrease in ev-T-PSA was not significant (p=0.055) (Figure 1B) and neither was the increase in T-PSA ev/srm ratio (p=0.078) (Figure 1C).

Following their response to the different treatments and subsequent improvement in clinical outcomes, all patients relapsed. Srm-T-PSA levels increased significantly during tumor progression (median: 38.0 μg/L; Q1-Q3: 6.7–137.9 μg/L; p=0.008) (Figure 1A), and although ev-T-PSA did not change (p=0.055) (Figure 1B), T-PSA ev/srm ratio decreased significantly (median: 1.2 %; Q1-Q3: 0.9–1.5 %) compared to concentrations observed during clinical response (median: 1.7 %; Q1-Q3: 1.3–3.6 %; p=0.008) (Figure 1C).

When comparing the results at disease progression with baseline values, a significant decrease in T-PSA ev/srm ratio values was observed (p=0.037). However, there were no significant changes in srm-T-PSA and ev-T-PSA concentrations (p=0.625 and p=0.482, respectively).

**Figure 2:**
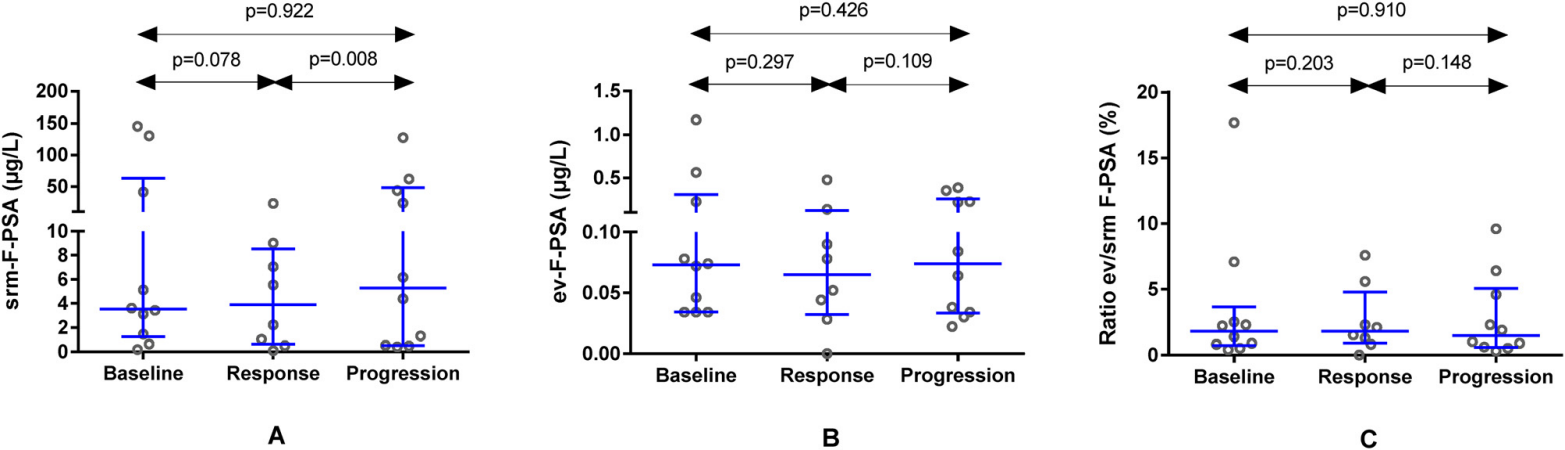
Comparative analysis of F-PSA concentrations in serum (srm−) (A) and extracellular vesicles (ev−) (B), and of F-PSA ev/srm ratio values (C) at baseline, during response to therapy and at disease progression in 10 patients with advanced prostate cancer. F-PSA, free PSA.

In the same patients’ samples, srm-F-PSA concentrations followed a similar trend to srm-T-PSA throughout the disease course (Figure 2A), although changes were only significant between therapy response and progression, with levels increasing from 3.9 μg/L (Q1-Q3: 0.6–8.5 μg/L) to 5.3 μg/L (Q1-Q3: 0.5–48.2 μg/L; p=0.008). In relation to ev-F-PSA (Figure 2B), and such as ev-T-PSA, changes were not significant at response (p=0.297) nor during progression (p=0.109). While the baseline F-PSA ev/srm ratio values increased at response and decreased at progression, as expected, the differences were not significant at any time during clinical evolution (p=0.203 and p=0.148, respectively) (Figure 2C).

In this case, no significant changes were observed when comparing progression to baseline values for srm-F-PSA, ev-F-PSA, and F-PSA ev/srm ratio (p=0.922, p=0.426 and p=0.910, respectively).

### Serum and extracellular vesicles PSA analysis according to patient’s treatment

**Figure 3:**
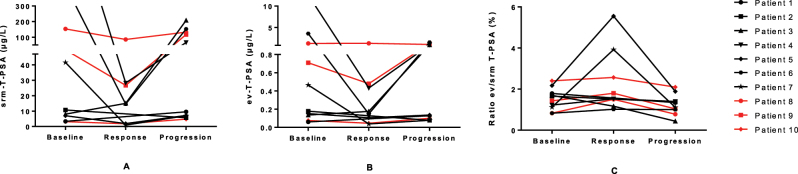
Representation of absolute changes in T-PSA concentrations in serum (srm−) (A) and extracellular vesicles (ev−) (B), as well as in T-PSA ev/srm ratio values (C) during response to therapy and at disease progression in 10 patients with advanced prostate cancer. T-PSA, PSA total.

**Figure 4:**
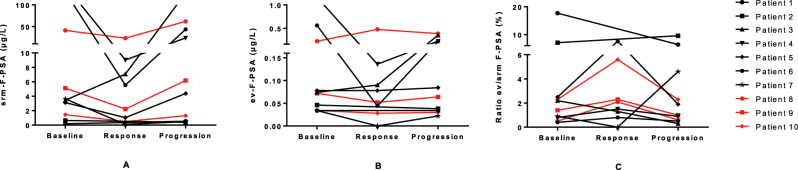
Representation of absolute changes in F-PSA concentrations in serum (srm−) (A) and extracellular vesicles (ev−) (B), as well as in F-PSA ev/srm ratio values (C) during response to therapy and at disease progression in 10 patients with advanced prostate cancer. F-PSA, free PSA.

The results for each patient were analyzed in order to investigate whether a relationship existed between changes in ev-PSA and the type of treatment received (Figures 3 and 4). Patients 1–7 received hormonal therapy, while patients 8–10 were treated with chemotherapy.

It was observed that all patients followed the same trend at response to treatment: a decrease in srm-T-PSA and ev-T-PSA concentrations, along with an increase in the T-PSA ev/srm ratio values; except patient 3 who exhibited the opposite behavior (Figure 3). As expected, during disease progression, srm-T-PSA and ev-T-PSA concentrations increased, while the T-PSA ev/srm ratio decreased respect to response in every patient except patient 8, in whom ev-T-PSA also decreased during progression. In addition, changes in ev-T-PSA upon progression were generally much less remarkable in those with lower srm-T-PSA concentrations. When comparing concentrations at progression to baseline values, 40 % of patients experienced an increase in srm-T-PSA and ev-T-PSA, 50 % had a decrease, and one patient (patient 5) showed a slight increase in srm-T-PSA and a decrease in ev-T-PSA. T-PSA ev/srm ratio decreased in 80 % of the participants, with patient 4 and patient 6 being the exceptions.

Among all participants, the best biochemical responses, with the greatest decreases in both srm-T-PSA and ev-T-PSA concentrations, were observed in three patients who received hormonal therapy: patient 4 (97.3 and 96.6 %, respectively), patient 6 (96.6 and 95.8 % respectively) and patient 7 (97.7 and 91.8 % respectively). The largest increase in the T-PSA ev/srm ratio was seen in patient 5 (156 %) and patient 7 (250 %). During progression, the most significant change in srm-T-PSA concentrations compared to response was also observed in patients undergoing hormonal therapy: patient 3 (1,282 %), patient 6 (947 %) and patient 7 (625 %). It should be noted that patient 6 also experienced the greatest increase in ev-T-PSA, so that his ratio remained constant during progression. On the other hand, in those patients treated with chemotherapy (patient 8, patient 9 and patient 10), less variation in serum and EVs PSA concentrations was observed, although they still followed the expected trend according to the disease course. The exception was patient 8, whose ev-T-PSA concentrations did not decrease during response, remaining constant.

Srm-F-PSA and ev-F-PSA concentrations, as well as F-PSA ev/srm ratio values, followed a similar trend to T-PSA throughout the course of the disease in nearly all patients (Figure 4). The exceptions were patient 7, whose F-PSA ev/srm ratio, in contrast to T-PSA, decreased at response and then increased markedly during progression, and patient 2, where discrepant results were observed between T-PSA and F-PSA ev/srm ratio.

## Discussion

Monitoring tumor response to treatment and early detection of relapses are of utmost importance in PCa, as recurrence of the disease, even several years after cure, is not uncommon. PSA has been used as a progression biomarker [10], being considered a reliable marker for recurrence and/or the emergence of new metastases [13], 14]. Furthermore, in advanced PCa, where treatments such as hormonal therapy, chemotherapy or immunotherapy may not completely eradicate the disease, PSA monitoring has been used to evaluate tumor response over time, determining treatment effectiveness, and deciding when to change therapy [18] (https://www.cancer.org/, accessed 21st June 2023). However, as previously described [19], 20], the use of serum PSA has notable limitations, prompting the need for alternative biomarkers that can provide specific and early insights into tumor tissue changes in response to the different systemic therapies.

In our study, the analysis of srm-T-PSA in sequential samples yielded results consistent with expectations: clinical improvement and response to treatment were associated with a significant decrease in serum tumor marker levels, which subsequently increased as the cancer progressed. Ev-T-PSA concentrations followed a similar trend; however, the changes were not significant at any time during the course of the disease. During tumor progression, T-PSA ev/srm ratio values decreased significantly compared to those observed at both the clinical response and baseline time points. The decrease in T-PSA ev/srm ratio values during progression may support the hypothesis that PSA incorporation into EVs and its subsequent release from the prostate into the circulation is more complex than that of soluble PSA. This process depends on production and structural tissue integrity, which may be modulated by the tumor microenvironment and cellular activity [28]. Thus, during disease progression most of the PSA produced by prostate tumor tissue would be released into the circulation in its soluble form, explaining the significant increase in srm-T-PSA, but not in ev-T-PSA concentrations. As a consequence, a decline in T-PSA ev/srm ratio was observed. Furthermore, these differences in the release of both molecular forms of PSA became more pronounced as srm-T-PSA concentrations increased. In general, in those samples with lower srm-T-PSA levels, the proportion of PSA released in EVs was more similar to soluble PSA, and the T-PSA ev/srm ratio values were higher. These findings correspond with previous observations reported by our group [31], where the T-PSA ev/srm ratio was notably higher in patients with srm-T-PSA <4 μg/L than those exceeding that cut-off. This suggests that the T-PSA ev/srm ratio could serve as a valuable tool for early detection of tumor progression, as exemplified in the results from patient 2. In this patient, the T-PSA ev/srm ratio decreased during progression, while PSA concentrations in serum and EVs did not increase as expected and, therefore, may not provide clear information about his clinical situation. It should be noted that this patient had the longest interval between baseline and post-progression samples, suggesting that this new biomarker may be particularly useful in such cases.

On the other hand, at clinical response to therapy, despite a decrease in srm-T-PSA concentrations, there was no significant decrease in ev-T-PSA, nor was there an increase in the T-PSA ev/srm ratio. These findings suggest that PSA bound to EVs, once released, remains in circulation longer than soluble PSA, implying a longer plasma half-life. Consequently, the decrease in ev-PSA concentrations would not be as significant as that of soluble PSA at the same time point of analysis. This is exemplified in the results of patient 8, where, despite srm-T-PSA decreased during clinical response, ev-T-PSA remained constant, leading to an increase in the T-PSA ev/srm ratio. It would be interesting to further explore this hypothesis by investigating the elimination kinetics of the EVs-bound PSA, in order to better characterize this novel form of PSA and clarify its distinct role compared to soluble PSA in tumor progression and clinical monitoring. Regarding F-PSA results, no significant changes were observed at any time, indicating that neither ev-F-PSA concentrations nor the F-PSA ev/srm ratio would be suitable alternatives as PCa monitoring biomarkers during the course of the disease.

When analyzing patients’ results according to the type of therapy received, we observed that patients who underwent hormonal therapy experienced greater variations in serum PSA concentrations, both at response and during progression, compared to those treated with chemotherapy. However, no significant association between treatment type and analytical changes during progression was observed in ev-T-PSA results, although patients treated with hormone therapy seemed to experience a greater decrease in ev-T-PSA at response. In terms of the T-PSA ev/srm ratio, no association was observed between the treatment received and changes in the ratio at any point during the disease course.

Our results serve as a proof of concept conducted on a small cohort of patients with advanced PCa and heterogeneous treatment regimens, which constitute the main limitations of the study. To gain a deeper understanding of the potential usefulness of ev-PSA in monitoring responses to different therapies and detecting tumor progression, the first and most essential step would be to recruit a larger cohort of patients, as this would enhance both the statistical power and the generalizability of the findings. Additionally, ensuring homogeneous patient populations based on the type of therapy received is crucial, as it minimizes confounding variables, reduces bias, and enables more precise and robust conclusions. Furthermore, expanding the scope to include treatment modalities such as immunotherapy – an increasingly important field and the foundation of numerous ongoing clinical oncology trials – could offer significant added value. For this reason, designing prospective studies that meet the outlined criteria would help validate our findings and strengthen their clinical relevance. Standardizing sample collection, processing, and storage protocols would further help minimize variability across patient samples, ensuring greater data consistency, and, consequently, more reliable conclusions regarding the clinical utility of ev-PSA in PCa follow-up. Finally, integrating additional biomarkers – such as other EV-associated proteins or RNA markers – and exploring correlations between prostate-derived EVs cargo and imaging techniques like PET-PSMA could provide a more comprehensive understanding of tumor progression and may be of particular interest for future research.

In conclusion, based on our findings, the T-PSA ev/srm ratio may serve as a valuable indicator of tumor progression and could be useful for detecting relapses in advanced PCa patients. However, neither ev-T-PSA nor T-PSA ev/srm ratio would be suitable as follow-up biomarkers for clinical response to hormonal treatments or chemotherapy.
